# 1 year mortality after hip fracture in an Irish urban trauma centre

**DOI:** 10.1186/s12891-023-06605-5

**Published:** 2023-06-13

**Authors:** Helena Ferris, Georgia Merron, Tara Coughlan

**Affiliations:** 1 Department of Public Health, Health Service Executive - South, Cork, Ireland; 2 Department of Age-Related Health Care, Tallaght University Hospital, Dublin, Ireland; 3 Discipline of Medical Gerontology, Trinity College Dublin, Dublin, Ireland

**Keywords:** Hip fracture, 1-year mortality, 1-year survival, Long-term outcomes, Irish hip fracture standards

## Abstract

**Background:**

Hip fracture accounts for a considerable burden of disease in older adults, yet there is a paucity of data pertaining to longer-term outcomes in the Irish Hip Fracture population. Understanding the factors that influence longer-term survival would allow care pathways to be refined to optimise patient outcomes. In Ireland, there is no linkage to death registration at a national or regional level, nor are longer-term outcomes captured by the Irish Hip Fracture Database. This study aimed to quantify 1-year mortality in an Irish hip fracture cohort and identify factors that influence survival at 1 year.

**Methods:**

A retrospective review of hip fracture cases in an Irish urban trauma centre over a 5-year period was conducted. Mortality status was obtained via the Inpatient Management System and correlated with the Irish Death Events Register. A range of routinely collected patient and care process variables were analysed using logistic regression.

**Results:**

A total of 833 patients were included. Within 1 year of sustaining a hip fracture, 20.5% (171/833) had died. On multivariate analysis, female gender (OR 0.36, p < 0.001, 95% CI 0.23–0.57), independent mobility pre-fracture (OR 0.24, p < 0.001, 95% CI 0.14–0.41) and early mobilisation on the day of or after surgery (OR 0.48, p < 0.001, 95% CI 0.30–0.77) reduced the likelihood of dying within 1 year (AUC 0.78).

**Conclusion:**

Of the variables examined, early postoperative mobilisation was the only modifiable factor identified that conferred a longer-term survival benefit. This underscores the importance of adhering to international best practice standards for early postoperative mobilisation.

**Supplementary Information:**

The online version contains supplementary material available at 10.1186/s12891-023-06605-5.

## Key Summary Points

**Aim**: To quantify 1-year mortality in an Irish hip fracture cohort and identify factors that influence survival at 1 year.

**Findings**: Within 1 year of sustaining a hip fracture, 20.5% of patients had died. Females who were independently mobile prior to fracture and mobilised on the day of or after surgery were less likely to die within 1-year.

**Take Home Message**: Of the variables examined, early postoperative mobilisation was the only modifiable factor identified that conferred a longer-term survival benefit.

**Supplementary Information:**

The online version contains supplementary material available at 10.1186/s12891-023-06605-5.

## Background

Older adults have a high risk of death after sustaining a hip fracture. Although geographical variations exist, approximately 15–29% of hip fracture patients die within 1 year of injury [1, 2]. With almost 3,700 hip fracture cases each year in Ireland, there is a need for robust data to identify survival trends and optimise the model of care [3].

Identifying predictors of longer-term mortality could help discriminate between those at higher risk of adverse outcomes and facilitate targeted interventions [4, 5]. Several factors have been shown to influence longer-term outcomes. A National Danish cohort study of > 113, 000 patients over a 15-year period demonstrated that male sex, increasing age, higher Charlson Comorbidity Index (CCI) and operation type other than total hip arthroplasty were independently associated with postoperative mortality [6]. These findings were similar to other European regions such as Spain where age > 80 years, male gender and CCI > 2 were also shown to be statistically significant predictors of 1-year mortality [7]. An Italian study by *Morri et al.* highlighted the importance of functional status and frailty, with lack of recovery of ambulation (OR 1.73, 95% CI 1.11–2.70) and activities of daily living scores (OR 1.25, 95% CI 1.14–1.38) being independent predictors of 1-year mortality after surgery [8]. Process measures also play a role as timely surgery within 48 h of admission has been shown to confer a survival benefit at 1 year (HR 0.83; 95% CI 0.82–0.85) [9].

In Ireland, the Irish Hip Fracture Database (IHFD) systematically collects data on hip fracture cases discharged from the acute hospital setting. The IHFD is a comprehensive data source, but like many national registries, it does not currently record longer-term outcome measures. The rationale for this study was to bridge the existing knowledge gap, as outcomes relating to longer-term mortality are under-reported in the Irish population. This study aimed to quantify 1-year mortality in a high-volume urban trauma centre and identify factors that influence survival at 1 year.

## National & International Landscape

There is a paucity of routinely reported long-term survival data across hip fracture registries globally. The Danish registry is one of the few national registries to report 1-year mortality on an annual basis, with 27.9% of patients dying in the first year following fracture [10]. A recent systematic review of 8 national registries and 36 different countries showed an overall mean 1-year mortality of 22% [11]. More specifically, reported 1 year mortality in Europe was 23.3%, Asia 17.9%, United States 21% and Australia 24.9% [11]. It is important to note that the patient population and healthcare systems vary considerably from country to country, which renders direct comparisons challenging [12]. In Ireland, national data pertaining to 1-year survival is not currently captured by the Irish Hip Fracture Database. However, a systematic review and meta-analysis of Irish literature reported a 1-year mortality of 24.2% [13].

## Methods

A retrospective review of all hip fracture cases over a 5-year period in Tallaght University Hospital (TUH) was conducted. TUH is one of 16 trauma centres nationally. It is a publicly funded level 4 hospital with a dedicated Orthogeriatric service since 2020. TUH admits over 1,500 trauma patients annually, a quarter of which are classified as major trauma cases [14]. It is a university teaching hospital with academic links to Trinity College Dublin.

### Inclusion criteria

All patients over 50 years discharged with hip fracture from TUH between 01/01/2017 and 31/12/2021 were included. Patients with a diagnosis of either hip fracture due to injury (ICD-10-AM diagnosis codes S72.00 to S72.2) or with a specified type of fracture (e.g., intracapsular displaced, intracapsular undisplaced, intertrochanteric, subtrochanteric or open) other than periprosthetic were included. Pathological fractures were not included. TUH submits data to the IHFD using a standardised data collection form. This captures evidence-based variables of interest ranging from patient demographics to pre-operative functional status and care process measures. Of note, early mobilisation by a Physiotherapist is one of the Irish Hip Fracture Standards and is defined as standing out of bed at a minimum on the day of or after surgery. Mortality status was obtained via the Inpatient Management System and correlated with the Irish Death Events Register.

### Analysis

Data were exported from Microsoft Excel into Stata® (version 17) for analysis. Descriptive statistics were used to describe the patient characteristics, in-hospital journey and outcomes. Continuous variables were expressed as mean (SD) or median (interquartile range [IQR]), while categorical data were expressed as numbers and percentages. Univariate logistic regression was undertaken to assess the impact of routinely collected variables on the likelihood of dying within 1 year of sustaining a hip fracture. A multivariate logistic regression model was constructed using the statistically significant variables from univariate analysis. Odds Ratios (OR) and 95% Confidence Intervals (CI) were used to describe the strength of the association. A value of p < 0.05 indicated statistical significance throughout. The discriminatory power of the model was expressed using Area Under the Curve (AUC) statistics.

## Results

### Descriptive statistics

This retrospective review included 833 hip fracture cases discharged from TUH from over a 5-year period. Data pertaining to 1-year mortality was obtained for 100% of cases. At 1 year post the date of admission, 20.5% (171/833) of patients had died. Mean Length Of Stay (LOS) was 22.3 days (SD 32.5). Females accounted for 67% (556/833) of the cohort with a mean age of 78.6 years (SD 8.9). Home was the most frequent source of admission. The most common fracture type was intertrochanteric (41.7%) with surgical repair carried out under Spinal Anaesthetic (SA) (46%). Table 1 outlines the characteristics of those who died within 1 year of sustaining a hip fracture.

**Table 1:** Characteristics of Patients Alive 1 Year After Hip Fracture Compared to Those Who Died

Description	Alive at 1 Year n (%)	Dead at 1 Year n (%)
**Mortality status, n (%)**	662/833 (79.5)	171/833 (20.5)
**Sex, n (%)**		
Female	463/662 (69.9%)	93/171 (54.3%)
Male	199/662 (30.1%)	78/ 171 (45.6%)
**Age group, n (%)**		
≥60	136/662 (20.5%)	11/171 (6.4%)
≥70	241/662 (36.4%)	50/171 (29.2%)
≥80	227/662 (34.3%)	76/171 (44.4%)
≥ 90	57/662 (8.6%)	32/171 (18.7%)
≥100	1/662 (0.2%)	2/171 (1.1%)
**Has medical card^, n (%)**		
No	200/662 (30.2%)	48/171 (28%)
Yes	462/662 (69.8%)	123/171 (72%)
**Trauma type, n (%)**		
High energy	40/662 (6.0%)	2/171 (1.1%)
Low energy	606/662 (91.6%)	164/171 (95.9%)
Not documented/other	16/662 (2.4%)	5/171 (2.9%)
**Fracture type, n (%)**		
Intertrochanteric	267/661 (40.3%)	80/171 (40.7%)
Intracapsular - displaced	202/661 (30.5%)	41/171 (23.9%)
Intracapsular - undisplaced	133/661 (20.1%)	33/171 (19.2%)
Subtrochanteric	29/661 (4.3%)	11/171 (6.4%)
Periprosthetic	20/661 (3.3%)	3/171 (1.7%)
Not documented/other	10/661 (1.5%)	2/171 (1.1%
**Admission source, n (%)**		
Home	566/662 (85.5%)	129/171 (75.4%)
Transfer from Acute Hospital	40/662 (6.1%)	9/171(5.3%)
Nursing Home/Conv. Home/Other Long-Stay	56/662 (8.4%)	33/171 (19.3%)
**Pre-fracture mobility, n (%)**		
Low functional mobility	237/574 (41.3%)	89/112 (79.5%)
High functional mobility (NMS > 6)^*^	337/574 (58.7%)	23/112 (20.5%)
**ASA grade, n (%)**		
Healthy (I)	14/636 (2.2%)	1/151 (0.6%)
Mild symptomatic (II)	264/636 (41.5%)	19/151 (12.6%)
Severe systematic (III)	294/636 (46.2%)	86/151 (56.9%)
Incapacitating systemic (IV)	30/636 (4.7%)	33/151 (21.9%)
Moribund (V)	0/636 (0%)	1/151 (0.6%)
Not documented	34/636 (5.3%)	11/151 (7.2%)
**Time to surgery, n (%)**		
No delay – operated on within 48 h	547/634 (86.3%)	115/150 (76.6%)
Awaiting inpatient or high-dependency bed	5/634 (0.7%	1/150 (0.6%)
Awaiting medical review/investigation	30/634 (4.7%)	20/150 (13.3%)
Awaiting orthopaedic diagnosis/intervention	12/634 (1.9%)	1/150 (0.6%)
Awaiting space on theatre list	3/634 (0.5%)	1/150 (0.6%)
Issues due to anticoagulation	4/634 (0.6%)	1/150 (0.6%)
Not documented/other	33/634 (5.2%)	11/150 (7.3%)
**Mobilised day of/after surgery, n/%**		
No	198/662 (29.9%)	91/171 (53.2%)
Yes	437/662 (66.0%)	60/171 (35.1%)
Not documented	27/662 (4.1%)	20/171 (11.7%)
**Acute Assessment by Geriatrician, n/%**		
No	176/661 (26.6%)	48/171 (28.0%)
Yes	484/661 (73.4%)	123/171 (72.0%)
**Admitted to Orthopaedic Ward, n/%**		
No	165/662 (24.9%)	50/171 (29.2%)
Yes	497/662 (75.1%)	121/171 (70.8%)

^A medical card allows patients under a specific income threshold to access primary care, community health services, dental services, prescription medicines and hospital care free of charge

*New Mobility Score (NMS) is a composite score of a patient’s ability to perform indoor walking, outdoor walking and shopping prior to hip fracture. Each parameter is scored between 0 and 3 (0: not at all, 1: with help from another person, 2: with an aid, 3: no difficulty) to give a maximum score of 9

### Analytical statistics

On univariate analysis, both patient and process factors influenced 1-year mortality in a statistically significant manner. Female gender (OR 0.51, p < 0.001, 95% CI 0.36–0.72), independent mobility pre-fracture (OR 0.18, p < 0.001, 95% CI 0.11–0.29) and early postoperative mobilisation (OR 0.29, p < 0.001, 95% CI 0.20–0.43) reduced the likelihood of death at 1 year (Table 2). Patients transferred from a Nursing Home were twice as likely to die within 1 year of fracture compared to those admitted from home (OR 2.5, p < 0.001, 95% CI 1.61–4.13).

**Table 2:** Univariate Logistic Regression

Variable	Odds Ratio	P Value	95% CI
**Sex**			
Male Female	1 (base) 0.51	0.00	0.36–0.72
**Age**			
50 60 70 80 90 100	1 (base) 0.07 0.20 0.33 0.56 1.99	0.07 0.26 0.44 0.68 0.71	0.00-1.27 0.01–3.37 0.02–5.41 0.03–9.28 0.05–78.24
**Medical Card**			
No Yes	1 (base) 1.10	0.58	0.76–1.61
**Pre-Fracture Mobility**			
Low functional mobility High functional mobility	1 (base) 0.18	0.00	0.11–0.29
**Mobilised Early**			
No Yes Unknown	1 (base) 0.29 1.61	0.00 0.13	0.20–0.43 0.85–3.02
**Fracture Type**			
Intertrochanteric Intracapsular – displaced Intracapsular – undisplaced Not documented/ other Periprosthetic Subtrochanteric	1 (base) 0.67 0.82 0.91 0.50 1.26	0.06 0.41 0.88 0.27 0.53	0.44–1.02 0.52–1.30 0.24–3.34 0.14–1.72 0.60–2.64
**Admission Source**			
Home Transfer from another hospital Transfer from nursing home	1 (base) 0.98 2.58	0.97 0.00	0.46–2.08 1.61–4.13
Length of Stay	1.01	0.00	1.00-1.01
**Surgical Delay**			
Awaiting inpatient or high-dependency bed Awaiting medical review/ investigation Awaiting orthopaedic diagnosis/ investigation Awaiting space on theatre list Issues due to anticoagulation No delay - surgery < 48 h Not documented/other	1 (base) 3.33 0.41 1.66 1.25 1.05 1.66	0.28 0.56 0.74 0.88 0.96 0.67	0.36–30.71 0.02–8.05 0.07–37.72 0.05–26.8 0.12–9.08 0.17–15.85
**Acute Assessment by Geriatrician**			
No Yes	1 (base) 0.92	0.70	0.63–1.35
**Admitted to Orthopaedic Ward**			
No Yes	1 (base) 0.80	0.25	0.55–1.16

On multivariate analysis, 3 variables remained statistically significant predictors of 1-year mortality (Table 3). Female gender (OR 0.36, p < 0.001, 95% CI 0.23–0.57), independent mobility prior to fracture (OR 0.24, p < 0.001, 95% CI 0.14–0.41) and early mobilisation on the day of or after surgery (OR 0.48, p < 0.001, 95% CI 0.30–0.77) reduced the likelihood of dying within 1 year of fracture.

**Table 3:** Multivariate Logistic Regression

Variable	Odds Ratio	P Value	95% CI
**Sex**			
Female	0.36	0.00	0.23–0.57
**Pre-Fracture Mobility**			
High functional mobility	0.24	0.00	0.14–0.41
**Mobilised Early**			
Yes No	0.48 1.52	0.00 0.41	0.30–0.77 0.55–4.19
**Admission Source**			
Transfer from another hospital Transfer from nursing home	0.63 1.46	0.42 0.22	0.20–1.94 0.78–2.74
Length of Stay	1.00	0.00	1.00-1.01

**Fig. 1:**
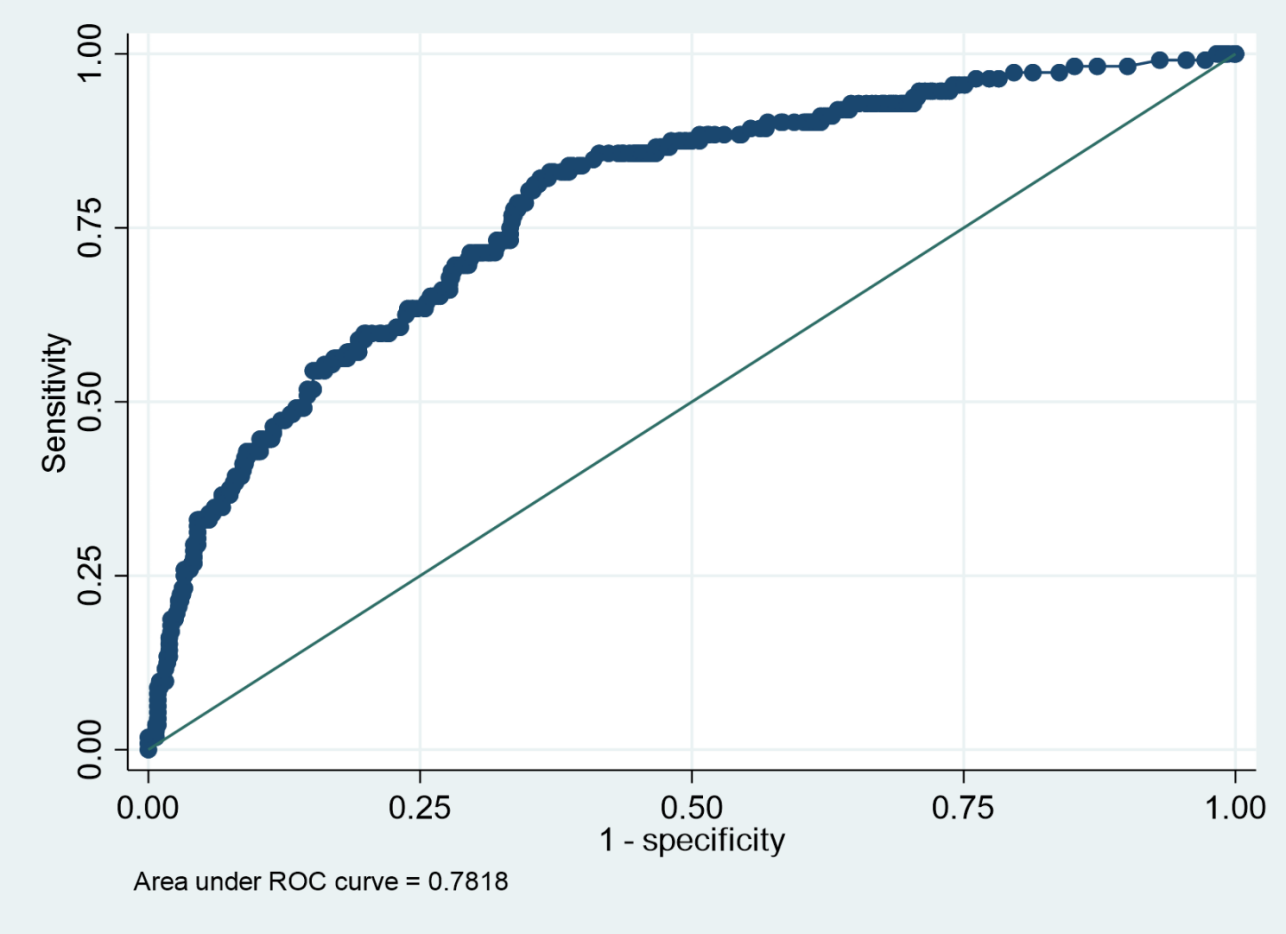
Area Under Curve Analysis

The model had a good discriminating ability with an AUC of 0.78 (Fig. 1).

## Discussion

Hip fracture can be a life-limiting event for older adults with 1-year mortality remaining high, despite advances in patient care. This study showed that 20.5% of patients died within 1 year of sustaining a hip fracture. This is lower than the recently reported systematic review and meta-analysis of Irish research, which showed a 1-year mortality of 24.2% and is also lower than the reported European mean 1-year mortality of 23.2% [11, 13].

The analysis presented in this paper showed that female gender, independent mobility prior to fracture and early mobilisation on the day of or after surgery reduced the likelihood of dying within 1 year of fracture. Of the variables examined, early mobilisation was the only modifiable factor identified. Those mobilised on the day of or after surgery were 52% less likely to die within 1 year of fracture (OR 0.48, p < 0.001, 95% CI 0.30–0.77). Early postoperative mobilisation by a Physiotherapist (stand out of bed at minimum) is one of the Irish Hip Fracture Standards (IHFS No. 7) with an associated Best Practice Tariff. Early mobilisation also is a clinical care standard in countries such as Denmark, Scotland, Australia and New Zealand. Currently, 78% of Irish hip fracture patients across all 16 trauma units achieve this national standard [3]. Given the positive impact of early mobilisation on both in-hospital and longer-term mortality, every effort should be made to ensure even greater compliance with this quality standard [15–17].

The findings have implications for service planning as there is a need to ensure adequate staffing levels to facilitate early postoperative ambulation. A service audit conducted by the National Office of Clinical Audit in 2020 showed that several trauma centres do not routinely have Physiotherapists or Physiotherapist Assistants available at weekends [3]. Where necessary, other members of the multidisciplinary team may be in a position to facilitate early ambulation. Moving forward, increased emphasis should be placed on involving and educating both patients and caregivers on the importance of getting up and moving as soon as possible after surgery [18]. This could be done at time of admission or as part of a pre-operative Orthogeriatric review, supported by a written information leaflet.

Another key finding from this study is the protective effect of high functional mobility pre-fracture (OR 0.24, p < 0.001, 95% CI 0.14–0.41). This is similar to the findings of a systematic review and meta-analysis by *Smith et al.* who also found pre-fracture mobility to be a statistically significant predictor of 1-year mortality. More specifically, patients who were independently mobile pre-fracture had an 87% lower risk of death at 6 months and 1 year compared to those who required assistance (RR 0.13, p < 0.001, 95% CI: 0.05, 0.34) [19]. This may reflect the interplay between mobility and frailty, which has been shown to predict both 1-year and 8-year mortality [20, 21]. Similarly, *Stubbs et al.* showed a statistically significant association between poor mobility pre-fracture and a range of adverse outcomes including mortality, prolonged LOS, postoperative complications and an increased likelihood of being discharged to a care home [22]. In practice, the findings of this study serve to identify a vulnerable group of patients who may benefit from early Orthogeriatric assessment. Males with poor pre-fracture mobility who are not mobilised early in the postoperative period have an increased risk of 1-year mortality. Strong public health messaging is required to promote healthy ageing and encourage older adults to stay active. Physical activity has been shown to improve balance, muscle and bone strength, which in turn reduces the risk of falls and subsequent fracture [23].

The collection of data pertaining to longer-term outcomes is central to the advancement of hip fracture care in Ireland. Excess mortality in the short term is well documented. As life expectancy increases, we need a greater understanding of the factors that impact survival and quality of life long after patients are discharged from the acute hospital setting. In a study of > 122,000 older adults across Europe and the USA with a 12 year follow up, *Katsoulis et al.* demonstrated that excess mortality persists for life [24]. This is similar to the findings of *Tiihonen et al.*, who followed a hip fracture cohort in Finland over 14 years and showed that hip fracture patients had a lower relative survival than the general population [25]. This underscores the importance of monitoring longer-term outcomes so that clinical care can be optimised to impart a meaningful benefit to patients. Ireland faces considerable challenges in the routine collection of longer-term data in the absence of data linkage to death registration and without a unique health identifier. Investment in the required technological infrastructure could utilise data that already exists within different silos of the health system and form a more comprehensive picture of the patient journey. Utilising data in this manner could enhance patient care by pinpointing high-impact areas in the care pathway and identifying high-risk high-cost patients who may benefit from early intervention [26].

### Limitations

Although this is a single centre study that is observational in nature, the findings are valid and have implications for patient care and service planning. Prior to this, 1-year mortality and factors influencing longer-term survival were unknown for the study population. The patient cohort is from a single urban trauma centre, which is one of 16 trauma units nationally that contributes to the IHFD. The findings of this study are generalisable to the wider Irish hip fracture population as the case mix and care pathway are in keeping with that of the national hip fracture population, as reported by the Irish Hip Fracture Database annual report [3]. Finally, this paper focuses solely on 1-year mortality. Moving forward, it is essential that outcomes such as quality of life are incorporated into the data collection process.

## Conclusion

This study quantified 1-year mortality in an Irish hip fracture cohort and identified factors that impact longer term survival. The findings highlight the protective effect of female gender, pre-operative functional status and early postoperative mobilisation. Of the variables examined, early mobilisation was the only modifiable factor identified that impacted 1-year mortality. This reinforces the importance of best practice standards for early postoperative mobilisation in conferring a real benefit to patient outcomes.

## Electronic supplementary material

Below is the link to the electronic supplementary material.

Supplementary Material 1

## Data Availability

The dataset used and/or analysed during the current study is available from the corresponding author on reasonable request.
