# Supplementary material for: 1 year mortality after hip fracture in an Irish urban trauma centre
Source: BMC Musculoskelet Disord. 2023 Jun 13;24:487. doi: 10.1186/s12891-023-06605-5 (PMC10262488; doi:10.1186/s12891-023-06605-5)
Supplement: Supplementary file 1 — Supplementary Material 1 [file 12891_2023_6605_MOESM1_ESM.pdf]

Tallaght University Hospital | Ospidéal Ollscoile Thamhlachta

An Academic Partner of Trinity College Dublin

Ospidéal San Séamas — Seirbhís Dé agus A Phobail

Research Office

Project ID: 3777

Professor Tara Coughlan ,

TUH

Classified Date: 9 June 2023

Submission Number: 2828

Submission Title: Longer term patient outcomes and relationship to adherence to/compliance with hip fracture quality standards Longer term patient outcomes and relationship to adherence to/compliance with hip fracture quality standards

Submission Date: 08.Jun.2023 15:36

Dear Professor Coughlan,

On behalf of the Chair and members of the SJH/TUH Joint Research Ethics Committee I wish to inform you that the above named project has been classified as **CLINICAL AUDIT.** As such, the project is not subject to ethics review, and has been reviewed for classification purposes only.

Yours sincerely,

Dr Sadhbh O'Neill

Research Ethics & Clinical Trials Manager,

SJH/TUH Joint Research Ethics Committee

The SJH/TUH Joint Research and Ethics Committee operates in compliance with and is constituted in accordance with the European Communities (Clinical Trials on Medicinal Products for Human Use) Regulations 2004 & ICH GCP guidelines.
